# SiO*_x_*/SiN*_y _*multilayers for photovoltaic and photonic applications

**DOI:** 10.1186/1556-276X-7-124

**Published:** 2012-02-14

**Authors:** Ramesh Pratibha Nalini, Larysa Khomenkova, Olivier Debieu, Julien Cardin, Christian Dufour, Marzia Carrada, Fabrice Gourbilleau

**Affiliations:** 1 CIMAP UMR CNRS/CEA/ENSICAEN/UCBN, 6 Bd. Maréchal Juin, 14050 Caen Cedex 4, France; 2 CEMES/CNRS, 29 rue J. Marvig, 31055 Toulouse, France

**Keywords:** SiO_x_/SiN_y_, multilayers, Nd^3+ ^doping, photoluminescence, XRD, absorption coefficient, conductivity

## Abstract

Microstructural, electrical, and optical properties of undoped and Nd^3+^-doped SiO*_x_*/SiN*_y _*multilayers fabricated by reactive radio frequency magnetron co-sputtering have been investigated with regard to thermal treatment. This letter demonstrates the advantages of using SiN*_y _*as the alternating sublayer instead of SiO_2_. A high density of silicon nanoclusters of the order 10^19 ^nc/cm^3 ^is achieved in the SiO*_x _*sublayers. Enhanced conductivity, emission, and absorption are attained at low thermal budget, which are promising for photovoltaic applications. Furthermore, the enhancement of Nd^3+ ^emission in these multilayers in comparison with the SiO*_x_*/SiO_2 _counterparts offers promising future photonic applications.

**PACS: **88.40.fh (Advanced materials development), 81.15.cd (Deposition by sputtering), 78.67.bf (Nanocrystals, nanoparticles, and nanoclusters).

## Introduction

Silicon nanoclusters [Si-ncs] with engineered band gap [1] have attracted the photonic and the photovoltaic industries as potential light sources, optical interconnectors, and efficient light absorbers [2-5]. Multilayers [MLs] of silicon-rich silicon oxide [SiO*_x_*] alternated with SiO_2 _became increasingly popular due to the precise control on the density and size distribution of Si-ncs [6,7]. Moreover, the efficiency of light emission from SiO*_x_*-based MLs exceeds that of the single SiO*_x _*layers with equivalent thickness due to the narrower Si-nc size distribution. The ML approach is also a powerful tool to investigate and control the emission of rare-earth [RE] dopants, for example, Er-doped SiO*_x_*/SiO_2 _MLs [8]. It also allows us to control the excitation mechanism of the RE ions by adjusting the optimal interaction distance between the Si-ncs and the RE ions. However, achieving electroluminescence and hence extending its usage for photovoltaic applications are problematic due to the high resistivity caused by SiO_2 _barrier layers [9]. Hence, replacement of the SiO_2 _sublayer by alternative dielectrics becomes interesting. Due to the lower potential barrier and better electrical transport properties of silicon nitride [Si_3_N_4_] in comparison to SiO_2_, multilayers like SiO*_x_*/Si_3_N_4 _[10], Si-rich Si_3_N_4 _(SiN*_y_*)/Si_3_N_4 _[11], and Si-rich Si_3_N_4_/SiO_2 _[12] were proposed and investigated [13] for their optical and electrical properties.

In this letter, we investigate SiO*_x_*/SiN*_y _*MLs and compare them with the SiO*_x_*/SiO_2 _counterparts reported earlier [9,14]. We demonstrate that an enhancement in the conductive and light-emitting properties of SiO*_x_*/SiN*_y _*MLs can be achieved with a reduced thermal budget. We also report a pioneering study on Nd-doped SiO*_x_*/SiN*_y _*MLs. A comparison between the properties of Nd^3+^-doped SiO*_x_*/SiO_2 _and SiO*_x_*/SiN*_y _*MLs are presented, and we show the benefits of using SiN*_y _*sublayers to achieve enhanced emission from Nd^3+ ^ions.

### Experimental details

Undoped and Nd-doped 3.5-nm SiO*_x_*/5-nm SiN*_y _*(50 periods) MLs were deposited at 500°C on a 2-inch p-Si substrate by radio frequency [RF] magnetron co-sputtering of Si and SiO_2 _targets in hydrogen-rich plasma for the SiO*_x _*sublayers and a pure Si target in nitrogen-rich plasma for the SiN*_y _*sublayers. An additional Nd_2_O_3 _target was used to dope the SiO*_x _*and SiN*_y _*sublayers by Nd^3+ ^ions. More details on the growth process can be found elsewhere [15]. The excess Si content in the corresponding SiO*_x _*and SiN*_y _*single layers obtained from RBS studies are calculated to be 25 and 11 at.%, respectively (i.e., SiO_*x *= 1 _and SiN_*y *= 1.03_). Conventional furnace annealing under nitrogen atmosphere at different temperatures, *T*_A _= 400 to 1,100°C, and times, *t*_A _= 1 to 60 min, was performed on the MLs. X-ray diffraction analysis was performed using a Phillips XPERT HPD Pro device (PANalytical, Almelo, The Netherlands) with CuK_α _radiation (*λ *= 0.1514 nm) at a fixed grazing angle incidence of 0.5°. Asymmetric grazing geometry was chosen to increase the volume of material interacting with the X-ray beam and to eliminate the contribution of the Si substrate. Photoluminescence [PL] spectra were recorded in the 550- to 1,150-nm spectral range using the Triax 180 Jobin Yvon monochromator (HORIBA Jobin Yvon SAS, Longjumeau, Paris, France) with an R5108 Hamamatsu PM tube (Hamamatsu, Shizuoka, Japan). The 488-nm Ar^+ ^laser line served as the excitation source. All the PL spectra were corrected by the spectral response of the experimental setup. Top and rear-side gold contacts were deposited on the MLs by sputtering for electrical characterization. Current-voltage measurements were carried out using a SUSS Microtec EP4 two-probe apparatus (SUSS Microtec, Germany) equipped with Keithley devices (Keithly, Cleveland, OH, USA). Energy-filtered transmission electron microscopy [EFTEM] was carried out on a cross-sectional specimen using a TEM-FEG microscope Tecnai F20ST (FEI, Eindhoven, The Netherlands) equipped with an energy filter TRIDIEM from Gatan (Gatan, München, Germany). The EFTEM images were obtained by inserting an energy-selecting slit in the energy-dispersive plane of the filter at the Si (17 eV) and at the SiO_2 _(23 eV) plasmon energy, with a width of ± 2 eV.

## Results and discussions

### Effect of annealing on the PL

**Figure 1:**
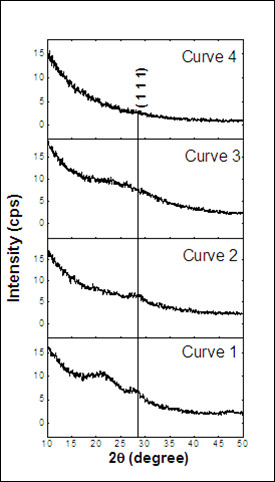
**XRD spectra of annealed Si-based MLs**. (curve 1) SiO*_x_*/SiO_2 _1 h, 1,100°C; (curve 2) SiO*_x_*/SiN*_y _*1 h, 1,100°C; (curve 3) SiO*_x_*/SiN*_y _*1 min, 1,000°C; and (curve 4) SiO*_x_*/SiO_2 _1 min, 1,000°C.

**Figure 2:**
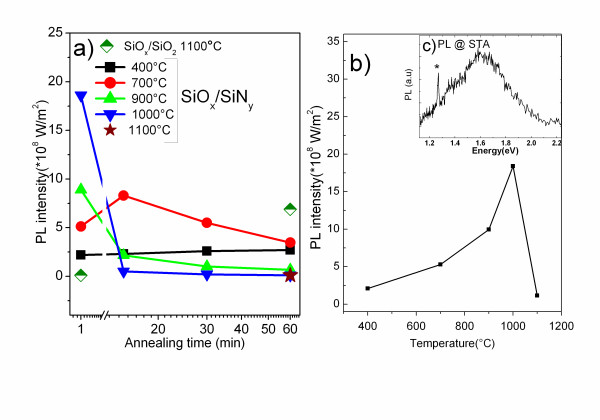
**Photoluminescence**. (**a**) Maximum PL intensity [*I*_PL_] of SiO*_x_*/SiN*_y _*MLs vs *T*_A _and *t*_A_, and SiO*_x_*/SiO_2 _at 1,100°C; (**b**) PL spectra of STA SiO*_x_*/SiN*_y _*MLs; (**c**) *I*_PL _vs *T*_A _for *t*_A _= 1 min. The asterisk represents the peak from second order emission of laser.

Since an annealing at *T*_A _= 1,100°C and *t*_A _= 60 min is the most suitable to achieve an efficient PL from Si-ncs either in sputtered SiO*_x _*single layers [7] or in SiO*_x _*/SiO_2 _MLs [16], such treatment was first employed on SiO*_x_*/SiN*_y _*MLs. The X-ray diffraction [XRD] broad peak centered around 2θ = 28° is the signature of the Si nanoclusters' formation in the SiO*_x _*/SiO_2 _(Figure 1, curve 1) and SiO*_x_*/SiN*_y _*MLs (Figure 1, curve 2) as already observed by means of atomic scale studies on similar multilayers [17]. However, contrary to the PL emission obtained from the SiO*_x_*/SiO_2 _MLs, no PL emission was observed in the SiO*_x_*/SiN*_y _*MLs after such annealing (Figure 2a). This stimulated a deeper investigation of the post-fabrication processing to achieve efficient light emission from the SiO*_x_*/SiN*_y _*MLs.

It was observed that the PL signals from the MLs annealed during *t*_A _= 60 min are significant only at lower temperatures (*T*_A _= 400°C to 700°C), and high intensities are obtained when the samples are annealed at high temperatures for a short time (*T*_A _= 900°C to 1,000°C, *t*_A _= 1 min). It is interesting to note that an interplay between *T*_A _and *t*_A _can yield similar PL efficiencies, as can be seen for *T*_A _= 900°C and *t*_A _= 1 min, and *T*_A _= 700°C and *t*_A _= 15 min (Figure 2a).

The highest PL intensity in SiO*_x_*/SiN*_y _*MLs was obtained with *T*_A _= 1,000°C and *t*_A _= 1 min (Figure 2b,c), whereas the SiO*_x_*/SiO_2 _MLs showed no emission after such short-time annealing treatment (Figure 2a). Corresponding XRD pattern of this short-time annealed [STA] (STA = 1 min, 1,000°C) SiO*_x_*/SiN_y _showed a broad peak in the range 2θ = 20° to 30° which is absent in STA SiO*_x_*/SiO_2 _MLs (Figure 1, curves 3 and 4). This suggests the presence of small Si clusters in the SiO*_x_*/SiN*_y _*MLs, with lower sizes (broader peak) by comparison with higher annealing temperature (1,100°C; Figure 1, curves 1 and 2). However, we cannot distinguish which of the sublayer is at the origin of the PL emission. Consequently, the recorded PL may be a combined contribution of the Si-ncs in the SiO*_x _*sublayers and the localized bandtail defect states in the SiN*_y _*sublayers.

### Absorption and electrical studies

**Figure 3:**
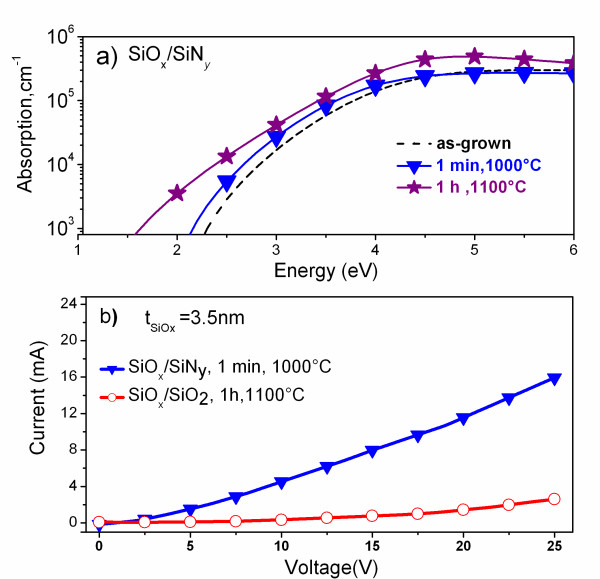
**Absorption coefficient and current-voltage behavior**. (**a**) Evolution of absorption coefficient with annealing; (**b**) Comparison of current-voltage behavior of SiO*_x_*/SiN*_y _*and SiO*_x_*/SiO_2 _MLs.

The absorption studies show similar absorption coefficients for as-grown and STA MLs, whereas annealing at *T*_A _= 1,100°C and *t*_A _= 60 min results in an absorption enhancement (Figure 3a). One can say that, at such temperature, an increase in density and size of the Si-ncs occurs due to phase separation of the SiO*_x _*sublayers into Si and SiO_2 _phases. The formation of Si nanocrystals is complete at *T*_A _= 1,100°C and *t*_A _= 60 min and leads to this enhancement. This reasoning is supported by the results obtained from the PL and the XRD analysis of the samples annealed at such temperature. The PL in the SiO*_x_*/SiN*_y _*MLs is quenched after an increase in the time and temperatures of annealing (Figure 2a), and this can be attributed to the increase in the size leading to the loss of quantum confinement effect. The formation of Si nanoclusters can be witnessed from the appearance of the XRD peak at 2θ = 28° (Figure 1, curve 2), which is not seen in the short-time annealed sample (Figure 1, curve 3).

Considering a balance between light emission and absorption for photovoltaic applications, we chose to study STA SiO*_x_*/SiN*_y _*MLs with a total thickness of 850 nm for electrical measurements. Figure 3b compares the dark current curves of 3.5-nm SiO*_x_*/5-nm SiN*_y _*with our earlier reported 3.5-nm SiO*_x_*/3.5-nm SiO_2 _(140 nm) MLs [14]. The resistivity was calculated at 7.5 V to be 2.15 and 214 MΩ·cm in the SiO*_x_*/SiN_y _and SiO*_x_*/SiO_2 _MLs, respectively. Since the thickness of the SiO*_x _*sublayer is the same in both cases (3.5 nm), this decrease in the resistivity of the SiO*_x_*/SiN_y _MLs can be ascribed to the substitution of 3.5-nm SiO_2 _by 5-nm SiN*_y _*sublayers. This hundred-times enhanced conductivity at low voltage paves way for further improvement of the SiO*_x_*/SiN*_y _*MLs' conductivity, for example, by decreasing the thickness of this SiN*_y _*sublayer.

### Microstructural studies

**Figure 4:**
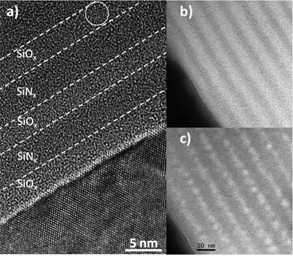
**HRTEM (a) and EFTEM (b, c) images**. SiO*_x_*/SiN*_y _*ML annealed at *T*_A _= 1,000°C, *t*_A _= 1 min by filtering the energy at SiO_2 _plasmon (b) and Si plasmon (c) energies, respectively.

The high-resolution transmission electron microscope [HRTEM] and EFTEM observations on STA SiO*_x_*/SiN*_y _*show Si-ncs in the SiO*_x _*sublayers with an average diameter of 3.4 nm. Only a couple of Si nanocrystals were observed in the HRTEM (Figure 4a), whereas a high density of Si-nanoclusters of about 10^19 ^nc/cm^3 ^can be witnessed from the EFTEM images taken at the Si plasmon energy (Figure 4c) implying that they are predominantly amorphous. Interestingly, this density of the Si-ncs in the SiO*_x_*/SiN*_y _*MLs is an order of magnitude higher than the Si-ncs formed in the SiO*_x_*/SiO_2 _MLs fabricated under similar conditions. The brighter SiO*_x _*sublayers are distinguished from the darker SiN*_y _*sublayers by filtering the SiO_2 _plasmon energy (Figure 4b). No evidence of Si-ncs within the SiN*_x _*sublayers was obtained. The STA could favor the formation of Si-ncs only in SiO*_x _*and not in SiN*_y _*sublayers. This could be attributed to the different mechanism of Si-ncs formation in SiO*_x _*and SiN*_y _*in MLs as opposed to that in single layers [18] and/or the low Si-excess content in SiN*_y_*.

### Effect of Nd^3+^-doping

**Figure 5:**
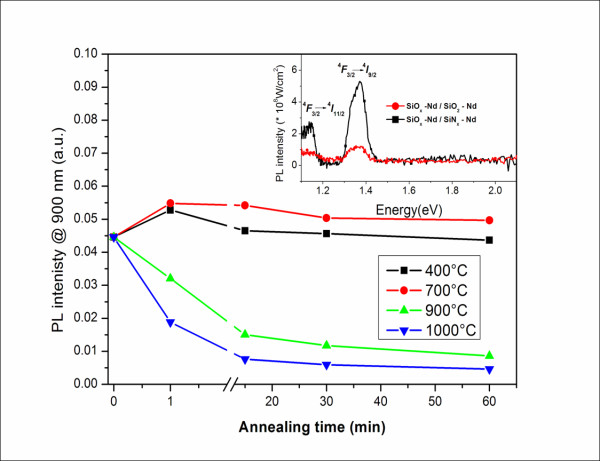
**PL intensity with annealing time and temperature**. Evolution of the Nd^3+ ^PL intensity at 1.37 eV for doped SiO*_x_*/SiN*_y _*MLs with annealing temperature and time. (Inset) PL spectra of as-grown Nd^3+^-doped SiO*_x_*/SiN*_y _*and SiO*_x_*/SiO_2 _MLs with equal number of periods. The thicknesses of the SiO*_x_*, SiO_2_, and SiN*_y _*sublayers are 3.5, 5.0, and 5.0 nm, respectively.

Understanding the microstructure of MLs and considering the enhancement of absorption and emission properties in SiO*_x_*/SiN*_y _*MLs compared to the SiO*_x_*/SiO_2 _MLs, we investigate the effect of using SiN*_y _*sublayer on the PL emission from Nd^3+ ^ions. For this purpose, the SiO*_x_*-Nd/SiN*_y_*-Nd and SiO*_x_*-Nd/SiO_2_-Nd MLs were fabricated, and their PL properties were compared. No PL emission was detected from the Nd^3+^-doped SiN*_y _*single layers at the different annealing treatments investigated here. Figure 5 shows the PL spectra of the Nd^3+^-doped as-grown MLs under non-resonant excitation with peaks corresponding to the ^4^*F*_3/2_→^4^*I*_9/2 _and ^4^*F*_3/2_→^4^*I*_11/2 _transitions at 1.37 and 1.17 eV, respectively. The comparison between the PL properties of undoped (Figure 2c) and Nd^3+^-doped MLs (Figure 5, inset) clearly shows the quenching of visible PL emission and the appearance of two Nd^3+^-related PL peaks in the Nd-doped MLs. Moreover, the intensity of Nd^3+ ^PL from the doped SiO*_x_*/SiN*_y _*MLs exceeds that of the SiO*_x_*/SiO_2 _MLs (Figure 5, inset). Thus, we deal with the efficient energy transfer towards Nd^3+ ^ions not only in SiO*_x _*but also in SiN*_y _*sublayers. Since this emission is observed for as-grown MLs, when no Si-ncs were formed in these MLS, it is obvious that the emission from the Nd^3+ ^ions in the SiN*_x_*-Nd sublayers is due to an efficient energy transfer from SiN*_y_*-localized defect states towards the Nd^3+ ^ions [19,20]. PL observed from the doped MLs after STA was not intense, and it was quenched with increasing annealing time. The same behavior was observed for the 900°C annealing. This could be due to the decrease in the number of defect-related sensitizers in SiN*_y _*and the formation of Nd_2_O_3 _clusters in the SiO*_x _*sublayers [21]. On the other hand, annealing at *T*_A _= 400°C to 700°C, discussed above for the undoped SiO*_x_*/SiN_y _MLs, enhance Nd^3+ ^PL emission when applied to the doped counterparts (Figure 3). Thus, we attain intense PL at a low thermal budget with *T*_A _(400°C to 700°C) and *t*_A _(1 min). To optimize Nd^3+ ^emission, the effect of the thickness of each sublayer in SiO*_x_*/SiN*_y _*MLs is under consideration now.

## Conclusion

In conclusion, we show that SiO*_x_*/SiN*_y _*MLs fabricated by RF magnetron sputtering can be engineered as structures for photovoltaic and photonic applications. The as-grown and STA SiO*_x_*/SiN*_y _*MLs show enhanced optical and electrical properties than the SiO*_x_*/SiO_2 _counterparts. Besides achieving a high density of Si-ncs at a reduced thermal budget, we show that high emission and absorption efficiencies can be achieved even from amorphous Si-ncs. The Nd-doped MLs, as-grown and those annealed at lower thermal budgets, demonstrate efficient emission from rare-earth ions. We also show that our STA SiO*_x_*/SiN*_y _*MLs have about a hundred times higher conductivity compared to the SiO*_x_*/SiO_2 _MLs. These results show the advantages of SiO*_x_*/SiN*_y _*MLs as materials for photovoltaic and photonic applications and open up perspectives for a detailed study.

## Abbreviations

MLs: multilayers; PL: photoluminescence; Si-nc: silicon nanoclusters; SiN*_y_*: silicon-rich silicon nitride; SiO*_x_*: silicon-rich silicon oxide; STA: short time annealing at 1,000°C for 1 min.

## Competing interests

The authors declare that they have no competing interests.

## Authors' contributions

RPN fabricated the undoped multilayers under investigation and carried out the characterization studies. LK and OD fabricated the Nd-doped layers and studied the effect of Nd doping on the MLs. JC and CD made contributions to the optical studies. MC performed the EFTEM measurements. FG conceived of the study and participated in the coordination of the manuscript. All authors read and approved the final manuscript.
